# Transition of Dephospho-DctD to the Transcriptionally Active State via Interaction with Dephospho-IIA^Glc^

**DOI:** 10.1128/mbio.03839-21

**Published:** 2022-03-21

**Authors:** Sebin Kang, Kyu-Ho Lee

**Affiliations:** a Department of Life Science, Sogang University, Seoul, South Korea; University of Washington

**Keywords:** biofilm, dephospho-DctD, dephospho-IIA^Glc^, exopolysaccharides, glucose

## Abstract

Exopolysaccharides (EPSs), biofilm-maturing components of Vibrio vulnificus, are abundantly produced when the expression of two major EPS gene clusters is activated by an enhancer-binding transcription factor, DctD_2_, whose expression and phosphorylation are induced by dicarboxylic acids. Surprisingly, when glucose was supplied to V. vulnificus, similar levels of expression of these clusters occurred, even in the absence of dicarboxylic acids. This glucose-dependent activation was also mediated by DctD_2_, whose expression was sequentially activated by the transcription regulator NtrC. Most DctD_2_ in cells grown without dicarboxylic acids was present in a dephosphorylated state, known as the transcriptionally inactive form. However, in the presence of glucose, a dephosphorylated component of the glucose-specific phosphotransferase system, d-IIA^Glc^, interacted with dephosphorylated DctD_2_ (d-DctD_2_). While d-DctD_2_ did not show any affinity to a DNA fragment containing the DctD-binding sequences, the complex of d-DctD_2_ and d-IIA^Glc^ exhibited specific and efficient DNA binding, similar to the phosphorylated DctD_2_. The d-DctD_2_-mediated activation of the EPS gene clusters’ expression was not fully achieved in cells grown with mannose. Furthermore, the degrees of expression of the clusters under glycerol were less than those under mannose. This was caused by an antagonistic and competitive effect of GlpK, whose expression was increased by glycerol, in forming a complex with d-DctD_2_ by d-IIA^Glc^. The data demonstrate a novel regulatory pathway for V. vulnificus EPS biosynthesis and biofilm maturation in the presence of glucose, which is mediated by d-DctD_2_ through its transition to the transcriptionally active state by interacting with available d-IIA^Glc^.

## INTRODUCTION

A major bacterial regulatory system in response to fluctuating environmental parameters is the two-component system (TCS), in which the sensor, a histidine protein kinase, transduces environmental signals to its cognate response regulator (1, 2). Most bacteria have numerous TCSs that control a wide range of cellular responses by regulating the expression or activity of their specific regulons via the transition of the response regulators between inactive and active states (3). The alteration of a response regulator to the active state is initiated once its cognate sensor kinase is autophosphorylated at a histidine residue upon exposure to specific environmental signals, such as various kinds of carbon, nitrogen, phosphorus, or sulfur sources (4–6). Phosphotransfer then rapidly occurs from the histidine residue in a sensor kinase to the aspartic acid residue in a response regulator. Phosphorylation usually induces a conformational change in response regulators, which endows the inactive regulators with the ability to perform specific outputs: for example, binding to the corresponding targets of DNA, RNA, or proteins (7, 8).

More than 65% of the so-far-identified response regulators are transcription factors that exhibit DNA-binding affinity if phosphorylated. Approximately 15% of them are categorized into the bacterial enhancer binding protein (bEBP) family, which is further subdivided into five groups based upon the structural characteristics in their N-terminal regulatory domains (9–13). In group I of bEBPs, which includes the well-known NtrC family, the transcriptionally active forms are phosphorylated oligomers, such as hexamers or heptamers. Regardless of the phosphorylation state of the monomeric form, the dimeric forms of bEBP are formed. However, further assembly to the oligomeric forms requires phosphorylation of the dimers, by which the AAA^+^ ATPase motifs located in the central domain of bEBP are conformationally opened to hydrolyze ATP. The produced hexamers or heptamers are then capable of binding to the upstream activator sequences for the target genes and interact with RpoN in the promoters via involvement of the C-terminal DNA-binding domain and the GAFTGA loop in the central domain of bEBP, respectively. ATP hydrolysis is necessary for the oligomeric forms of bEBP to initiate the transcription process by stabilizing these interactions (12).

DctD, belonging to group I bEBPs, has been extensively studied in nitrogen-fixing bacteria, in which the dicarboxylic acids transported from the host’s roots are the main sources of electrons, reducing dinitrogen to ammonia (14). Its sensor kinase, DctB, senses the ambient dicarboxylic acids and phosphorylates DctD, which in turn activates the transcription of genes encoding the uptake system(s) for dicarboxylic acids (15). In addition to nitrogen-fixing bacteria, many bacterial species, including the model foodborne pathogen Vibrio vulnificus, are also equipped with DctBD (16). In the genomes of V. vulnificus, two operons encoding the DctBD homologs, *dctB_1_D_1_* and *dctB_2_D_2_*, are present, but it was found that DctD_2_ is responsible for activating the transcription of exopolysaccharide (EPS) EPS-II and EPS-III clusters in the presence of fumarate (16). Notably, biosynthesis of EPSs, the loosely associated polysaccharides outside bacterial envelopes (17), is regulated at the transcription level via the involvement of DctD_2_, which activates the expression of EPS gene clusters in the presence of various kinds of dicarboxylic acids (16). Furthermore, the transcription of the *dctB*_2_*D*_2_ operon is activated by NtrB/C, the TCS responsive to the carbon/nitrogen ratio (16). The findings that the production and secretion of EPSs, which are presumed to be entirely composed of carbohydrates, are regulated by both NtrB/C and DctB_2_/D_2_ have implied the presence of a common regulatory connection or connections in bacterial recognition of the depletion of nitrogen sources and the repletion of some carbon sources.

A dramatic example of bacterial adaptational behavior at the population level is the formation of biofilms in response to various environmental conditions, which enhances the survival of individual cells under stress conditions, including the host environment (18, 19). EPSs are the most abundant constituents comprising the extracellular polymeric matrix (EPM) of biofilms and the highly active components interacting with other EPM components: e.g., proteins, polysaccharides, nucleic acids, and lipids (17, 20–25). Thus, EPSs are considered essential constituents for the construction of mature biofilms by facilitating the interactions of a bacterial cell with adjacent cells and substrates (26). V. vulnificus has a high risk of causing fatal septicemia or gastroenteritis (27); it has been shown that its ability to form mature biofilms is critically dependent upon the biosynthesis of EPSs (28). There are at least three gene clusters in V. vulnificus genomes: EPS-I (the *rbd* operon), EPS-II (the *brp* operon), and EPS-III (28–30). The EPS-II and EPS-III clusters are required for production of EPSs involved in biofilm maturation under the conditions containing dicarboxylic acids, although EPS compositions have not yet been elucidated in this bacterial species (28, 31). One of the major regulators for induction of these clusters has been shown to be DctB/D (16).

The abilities of V. vulnificus to form mature biofilms and to disperse cells from the robust biofilm structures have been correlated with its pathogenicity in an animal model (28, 31, 32). As shown in diverse pathogenic bacteria (33, 34), it is assumed that the assembly of biofilms and the dispersal from these structures would provide V. vulnificus with the advantages of survival and proliferation in host environments. Therefore, appropriate expression of EPS gene clusters is required for this foodborne pathogen upon its sensing host-specific environments, such as carbon sources that are relatively abundant in host environments. Among the various available carbon sources, glucose is abundantly present in various biofluids, tissues, and organs of humans (35, 36), and its uptake and utilization are controlled by the phosphotransferase system (PTS) and diverse regulatory mechanisms, including carbon catabolite repression (37, 38).

**FIG 1:**
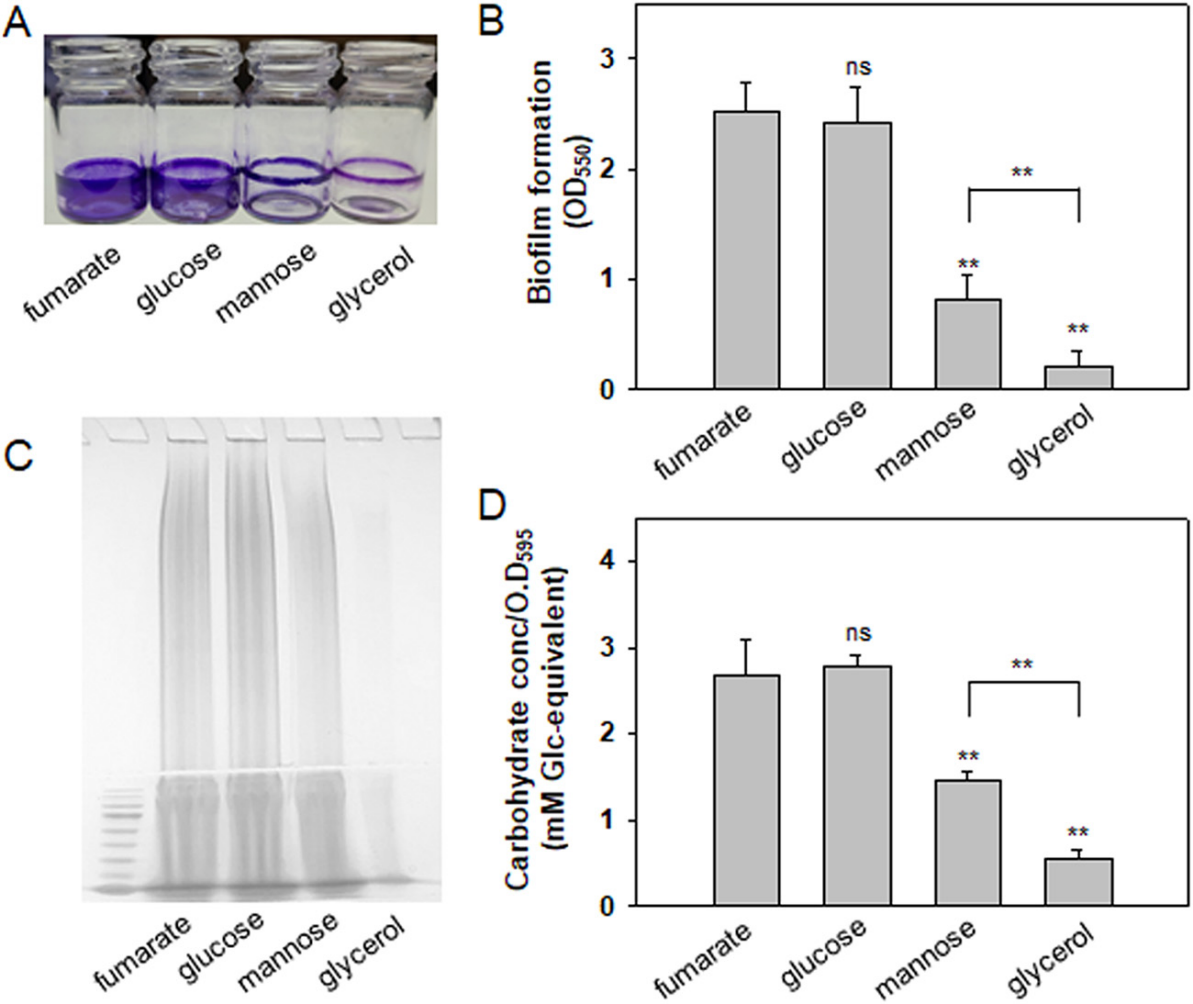
Biofilm formation and EPS production by V. vulnificus in the presence of fumarate, glucose, mannose, or glycerol. (A and B) Biofilm formation. Wild-type V. vulnificus cells were statically incubated for 48 h in AB medium supplemented with fumarate, glucose, mannose, or glycerol. The biofilms that developed on the borosilicate tubes were estimated by staining with crystal violet (A). The associated dyes were dissolved and measured by spectrophotometry at 550 nm (B). The *P* values for comparison with the AB-fumarate incubation are indicated (**, *P* < 0.001; *, 0.001 ≤ *P* < 0.01; ns, not significant). A comparison between AB-mannose and AB-glycerol incubations was also included with a *P* value above a corresponding horizontal line. (C and D) EPS production. Wild-type V. vulnificus cells grown on the AB agar plates supplemented with fumarate, glucose, mannose, or glycerol, were subjected to the process for EPS extraction, as previously described (28). The resultant EPS extracts run in a 5% stacking polyacrylamide gel were visualized by staining with Stains-All (C). The carbohydrate contents in each extract were measured by the phenol-sulfuric acid method (64). The estimated carbohydrate contents were expressed as mM glucose equivalents per cell masses equivalent to an OD_595_ of 1.0 (D). *P* values are presented as described above.

In our preliminary investigation to screen the factors inducing biofilm formation of V. vulnificus, it was noticed that glucose was able to induce biofilm maturation via increasing the biosynthesis of EPSs by virtue of the transcription factor DctD_2_ (shown in Fig. 1), similar to dicarboxylic acids. Interestingly, DctD_2_ is presumed to be present in an unphosphorylated, inactive form in the absence of dicarboxylic acids. Thus, in this study, we further investigated the regulatory mechanism that activates the expression of EPS gene clusters in the presence of glucose, which mimics the regulatory role of the phosphorylated form of DctD_2_.

## RESULTS

### Biofilm formation and EPS production by V. vulnificus were highly induced in the presence of glucose.

It has been previously shown that V. vulnificus produces significantly increased sizes of biofilms under growth conditions supplemented with dicarboxylic acids, such as fumarate, compared to biofilms formed in the presence of glycerol (16). In this study, to further examine the effects of other carbon sources on biofilm formation by V. vulnificus, the wild-type (WT) strain of this bacterium was incubated for 48 h in AB medium (defined in Materials and Methods) containing PTS sugars, such as glucose and mannose. The resultant biofilms were compared with those formed in AB-fumarate or AB-glycerol (Fig. 1A). In the presence of glucose, the extent of biofilm formation was almost the same as that in the presence of fumarate. In contrast, biofilms formed in AB-mannose were intermediate between those in AB-fumarate and AB-glycerol (Fig. 1B): The biofilms formed in AB-mannose were estimated to be 0.3× and 3.8× the sizes of the biofilms formed in AB-fumarate and AB-glycerol, respectively.

The differences in biofilm formation were well correlated with the degree of EPS production by V. vulnificus cells in the presence of the same kinds of carbon sources supplied in the AB medium (Fig. 1C). The levels of EPS production by wild-type V. vulnificus cells grown in the presence of glucose were 1.0, 1.9, and 5.1 times higher than those by the cells grown in the presence of fumarate, mannose, and glycerol, respectively (Fig. 1D). These results imply that the biosynthetic factors for EPSs, the most critical components for biofilm maturation by V. vulnificus, were similarly expressed in V. vulnificus grown in the presence of fumarate or glucose.

### Increased production of EPS in the presence of glucose was due to the induced expression of EPS-II and EPS-III gene clusters.

**FIG 2:**
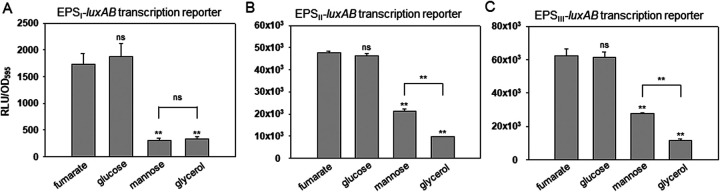
Expression of three EPS gene clusters of V. vulnificus in the presence of fumarate, glucose, mannose, or glycerol. Wild-type V. vulnificus cells carrying a *luxAB* transcriptional reporter fused with each EPS gene cluster (EPS-I [A], EPS-II [B], or EPS-III [C]) (28) were grown in AB-fumarate, -glucose, -mannose, or -glycerol medium supplemented with 3 μg/mL tetracycline. At an OD_595_ of 0.4, cells were harvested, and their luciferase activities were measured using a luminometer. The degree of cluster expression was expressed as a normalized value: the relative light unit (RLU) divided by the cell mass (OD_595_) of each sample. The *P* values are presented as described in the legend to Fig. 1.

Since EPS production is regulated at the transcriptional levels of three gene clusters for EPS biosynthesis in V. vulnificus (28), the effects of glucose and mannose on the expression of the EPS gene clusters were examined using V. vulnificus carrying a *luxAB* transcription reporter fused with the upstream region of each EPS cluster. All three EPS clusters were highly induced in AB-fumarate compared to AB-glycerol, as previously shown (16, 28). The clusters were also well expressed in AB-glucose, with almost the same degree of expression in AB-fumarate (Fig. 2A to C). In AB-mannose, the expression of the EPS-I cluster, which is directly activated by NtrC (16), was significantly decreased to basal levels, as shown in AB-glycerol (Fig. 2A). However, the levels of expression of EPS-II and EPS-III clusters in AB-mannose, which are directly activated by DctD_2_ (16), were significantly lower than those in AB-glucose, but higher than those in AB-glycerol (Fig. 2B and C). These results suggest that the significantly increased levels of biofilm formation and EPS production in AB-glucose (Fig. 1), compared to those in AB-mannose, were due to the increased transcription levels of the EPS gene clusters, possibly via the involvement of DctD even in the presence of glucose.

As previously reported (16), among two DctD homologs in V. vulnificus, only DctD_2_ has an amino acid residue (D57) that can be phosphorylated in the presence of dicarboxylic acids. Thus, DctD_2_ is a transcription factor responsible for activating the transcription of EPS-II and EPS-III clusters in the presence of fumarate (16). Since DctD cannot be phosphorylated without a dicarboxylic acid, the dephosphorylated forms of DctD, which could be either a dephosphorylated DctD_2_ or a naturally unphosphorylated DctD_1_, would activate the transcription of the clusters. Thus, to identify the functional DctD(s) in transcriptional activation of the gene clusters in the presence of glucose, V. vulnificus strains carrying *luxAB* transcription reporter fused with *dctA*, one of the representative genes belonging to the DctD regulon (15, 16), were grown in AB medium containing fumarate, glucose, mannose, or glycerol (see Fig. S1 in the supplemental material). Its expression in the Δ*dctD_1_* mutant was similar to that in the wild type. Furthermore, its expression patterns were similar to those of EPS-II and EPS-III clusters in the wild type: expression was high in AB-fumarate and AB-glucose, intermediate in AB-mannose, and basal in AB-glycerol. However, expression of *dctA* in the Δ*dctD_2_* mutant was minimal regardless of the carbon source in the growth medium, demonstrating that DctD_2_ is required for induction of the transcription of *dctA*.

10.1128/mbio.03839-21.1 FIG S1 Expression of *dctA* in the presence of fumarate, glucose, mannose, or glycerol. V. vulnificus wild-type and Δ*dctD_1_* and Δ*dctD_2_* mutant strains carrying the *luxAB* transcription reporter fused with the regulatory region of the *dctA* (16) were grown in the AB-fumarate, -glucose, -mannose, or -glycerol medium supplemented with tetracycline (3 μg/mL). At an OD_595_ of 0.4, cells were harvested, and their luciferase activities were measured using a luminometer. The degrees of *dctA* expression are expressed as a normalized value: the relative light unit (RLU) divided by the cell mass (OD_595_) of each sample. The *P* values for comparison with AB-fumarate incubation are indicated: **, *P* < 0.001; ns, not significant at *P* ≥ 0.01. Download FIG S1, TIF file, 1.7 MB. Copyright © 2022 Kang and Lee. 2022 Kang and Lee. https://creativecommons.org/licenses/by/4.0/ This content is distributed under the terms of the Creative Commons Attribution 4.0 International license.

### Both *ntrC* expression and *dctD* expression were induced by glucose.

**FIG 3:**
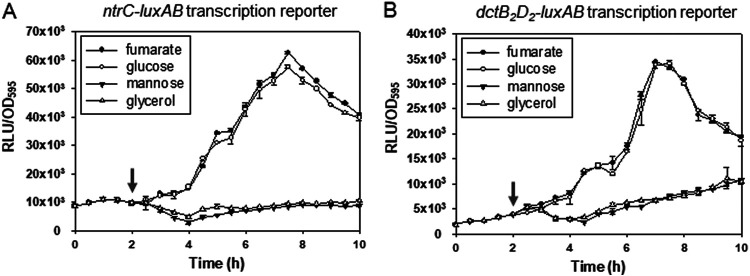
Expression of *ntrC* and *dctD_2_* genes of V. vulnificus in the presence of fumarate, glucose, mannose, or glycerol. Wild-type V. vulnificus cells carrying a *luxAB* transcriptional reporter fused with the regulatory region of the *glnA-ntrB-ntrC* operon (32) (A) or the regulatory region of the *dctB_2_D_2_* operon (16) (B) were grown for 2 h in AB medium supplemented with glycerol (22 mM) and tetracycline (3 μg/mL) and then subcultured to AB-fumarate, -glucose, -mannose, and -glycerol media supplemented with 3 μg/mL tetracycline. Bacterial cells were harvested at every 30 min, and light production was measured and presented as described in the legend to Fig. 2. The arrows indicate the point at which V. vulnificus cells pregrown in AB-glycerol were challenged with different carbon sources.

Since the maximal degrees of expression of the EPS-II and EPS-III clusters were observed in cells grown in the presence of fumarate or glucose (Fig. 2), the expression of DctD_2_ was examined by measuring the transcription of the genes encoding DctD_2_ as well as its transcription activator, NtrC. As it was previously reported that *ntrC* transcription was induced in the presence of dicarboxylic acids and then NtrC activated the *dctD_2_* transcription (16), whether glucose exhibited a similar effect on the expression of NtrC and DctD_2_ was investigated (Fig. 3). V. vulnificus carrying *luxAB* transcription reporters fused with DNA fragments upstream of the *glnA-ntrB-ntrC* operon or the *dctB_2_D_2_* operon were grown in AB-glycerol. After 2 h of incubation, fumarate, glucose, mannose, or glycerol was added to each culture. The basal levels of the *ntr*-reporter (Fig. 3A) and *dct_2_*-reporter (Fig. 3B) fusions in AB-glycerol for the first 2 h of incubation were highly induced by the addition of fumarate and glucose. In contrast, the addition of mannose did not result in the induction of either reporter, as shown by glycerol. These results showed that glucose induces the expression of both *ntrC* and *dctD_2_*. Since NtrC activates the transcription of *dctB_2_D_2_* operon, as previously reported (16), it is postulated that the induced NtrC activated *dctD_2_* transcription, and then the increased expression of DctD_2_ would subsequently result in the induced expression of EPS-II and EPS-III clusters in AB-glucose.

### The dephosphorylated form of DctD_2_ activated the transcription of the EPS-II and EPS-III gene clusters in the presence of the dephosphorylated form of IIA^Glc^.

**FIG 4:**
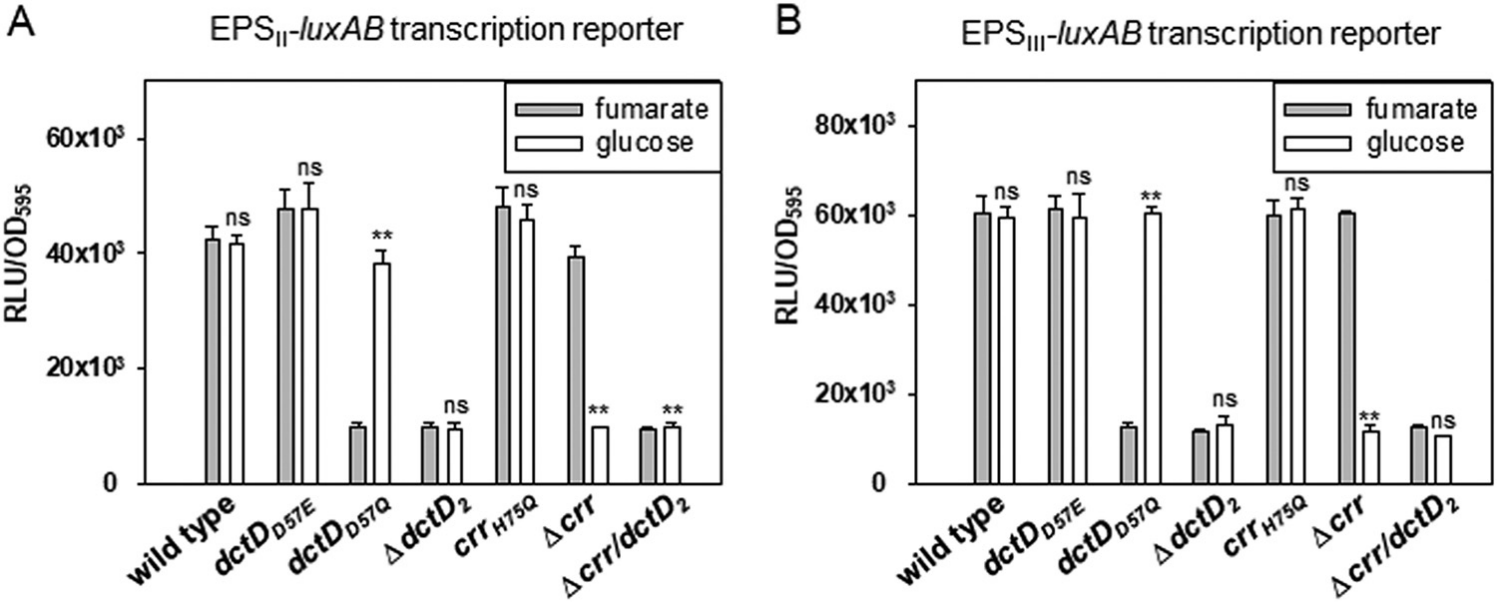
Expression of the EPS-II and EPS-III gene clusters in various mutant V. vulnificus strains defective in DctD_2_ and/or IIA^Glc^. V. vulnificus strains carrying mutant DctD_2_ (i.e., Δ*dctD*_2_, a phospho-form of DctD [*dctD*_D57E_], and a dephospho-form of DctD [*dctD*_D57Q_]), mutant IIA^Glc^ (i.e., Δ*crr* and a dephospho-form of IIA^Glc^ [*crr*_H75Q_]), or deletion of two ORFs (Δ*crr* Δ*dctD_2_*) were used to compare the expression of two EPS gene clusters. Each strain carrying either EPS-II (A) or EPS-III (B) was inoculated in AB-fumarate and AB-glucose media. After incubation for 4 h, the cell densities and light production were measured, and the extents of the gene clusters’ expression were presented as described in the legend to Fig. 2. The *P* values for comparison with the samples in fumarate and glucose are indicated: **, *P* < 0.001; ns, not significant.

Transcriptional activation of the EPS-II and EPS-III clusters occurs via phosphorylation of DctD_2_ by dicarboxylic acids, specifically fumarate (16). Thus, it is questionable how dephosphorylated DctD_2_ (d-DctD_2_) in cells grown in the presence of glucose could play a role in activating transcription as phosphorylated DctD_2_ (p-DctD_2_) in cells grown with fumarate. To address this issue, various strains of V. vulnificus mutated at the *dctD_2_* locus were utilized to monitor the expression of two *luxAB*-reporters. This experiment included the strains in which *dctD_2_* was deleted (Δ*dctD_2_*), substituted for with an open reading frame (ORF) encoding p-DctD_2_ (*dctD*_D57E_), and substituted for with an ORF encoding d-DctD_2_ (*dctD*_D57Q_). Each strain carrying a reporter of either EPS-II (Fig. 4A) or EPS-III (Fig. 4B) was inoculated in AB-fumarate and AB-glucose media, and their expression at the mid-exponential phase was determined. Basal levels of expression occurred in the Δ*dctD*_2_ mutant, which further supported the dependency of two clusters’ transcriptions on DctD_2_ under both incubation conditions. In the *dctD*_D57E_ strain, the expression of two clusters in either AB-fumarate or AB-glucose was at maximal levels, which was achieved by p-DctD_2_ regardless of the carbon sources in the growth medium. The expression of two reporters in the *dctD*_D57Q_ strain, however, was induced only in AB-glucose up to the expression levels in the *dctD*_D57E_ strain, suggesting that d-DctD_2_ plays a critical role in activating the clusters’ transcription in AB-glucose. The findings indicated that the transition of d-DctD_2_ into the transcriptionally active state was achieved in a glucose-specific manner.

Therefore, we hypothesized the involvement of a component that is able to specifically sense the presence of glucose and then endow transcription activity to d-DctD_2_. The mutants of V. vulnificus with mutation at the *crr* locus were then analyzed, including the strains carrying a deletion of IIA^Glc^ (Δ*crr*), a dephospho-form of IIA^Glc^ (*crr*_H75Q_), and deletions of both *crr* and *dctD_2_* ORFs (Δ*crr* Δ*dctD_2_*). The maximal and basal expression levels of the two clusters were obtained in the Δ*crr* mutant grown in AB-fumarate and AB-glucose, respectively, which indicated that the effect of *crr* deletion on the expression of the two clusters was only observed in the cells grown in AB-glucose. In addition, a double mutant produced basal levels of expression, which were the same levels as the Δ*dctD_2_* mutant. Thus, the glucose-specific component involved in the above hypothetical regulatory pathway is IIA^Glc^. Since the phosphorylated state of IIA^Glc^ is determined by glucose (39), it was assumed that d-IIA^Glc^ might influence the transcription activity of d-DctD_2_. To test this assumption, the expression of the two clusters was examined using *crr*_H75Q_. In AB-glucose, the *crr*_H75Q_ strain showed the maximal expression of two clusters, which were the same levels found in the *crr*_H75Q_ strain grown in AB-fumarate. These results suggested that d-DctD_2_ became a transcriptionally active state if the cellular levels of d-IIA^Glc^ increased under growth conditions containing glucose.

### Specific interaction of d-DctD_2_ with d-IIA^Glc^.

**FIG 5:**
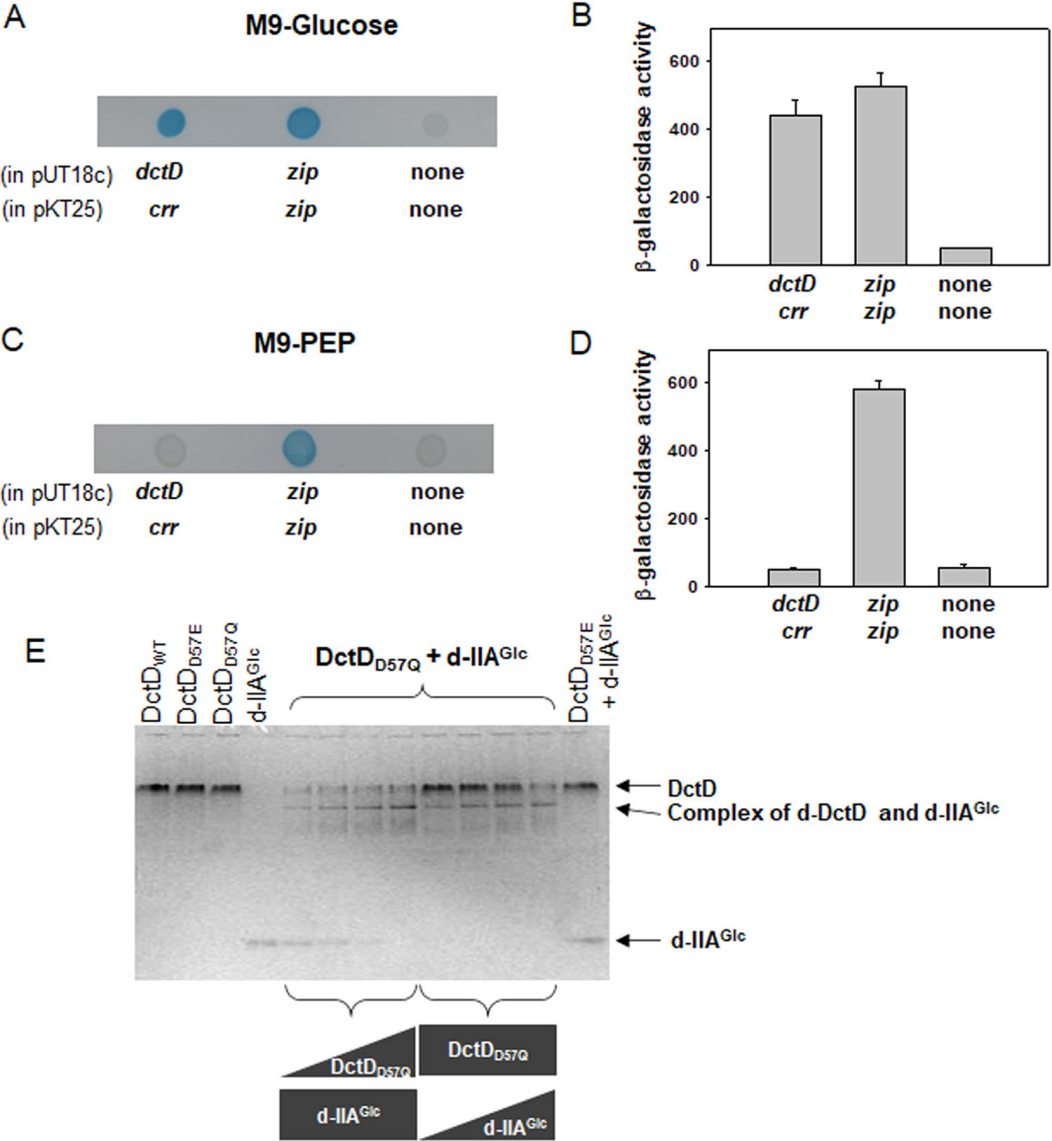
Specific interaction between DctD_2_ and IIA^Glc^ proteins. (A to D) Bacterial two-hybrid system. Two plasmids, pUT18c-*dctD_2_* and pKT25-*crr* (Table S1), were constructed and cotransformed to E. coli BTH101, as described in Materials and Methods. This transformant was grown in M9 medium supplemented with glucose (Glc) (A and B) or phosphoenolpyruvate (PEP) (C and D), and then the resultant β-galactosidase activities were examined. For comparison, the negative and positive controls (E. coli BTH101 harboring pUT18c/pKT25 and pUT18c-*zip*/pKT25-*zip*, respectively), were included in these assays: shown are blue colonies on the agar plates supplemented with 40 μg/mL X-Gal (A and C) and the specific β-galactosidase activities in Miller units (B and D), as described in Materials and Methods. (E) *In vitro* interaction between d-IIA^Glc^ and d-DctD_2_. To examine the role of the phosphorylated states of DctD_2_ in specific interaction with d-IIA^Glc^, both phosphorylated (p-) and dephosphorylated (d-) forms of recombinant DctD_2_ were prepared: the original DctD_2_ (DctD_WT_), p-DctD_2_ (DctD_D57E_), and d-DctD_2_ (DctD_D57Q_) (Table S1). Various combinations of DctD_2_ and d-IIA^Glc^ proteins were mixed, and the reaction mixtures were run in a native gel. Lane 1, DctD_WT_ (5 μM); lane 2, DctD_D57E_ (5 μM); lane 3, DctD_D57Q_ (5 μM); lane 4, d-IIA^Glc^ (5 μM); lanes 5 to 8, d-IIA^Glc^ (5 μM) with DctD_D57Q_ at a concentration of 0.04, 0.2, 1, or 5 μM; lanes 9 to 12, DctD_D57Q_ (5 μM) with d-IIA^Glc^ at a concentration of 0.04, 0.2, 1, or 5 μM; and lane 13, DctD_D57E_ (5 μM) with d-IIA^Glc^ (5 μM). Each protein band and the newly emerged band are indicated with arrows.

IIA^Glc^ interacts with various proteins and regulates the activities of the target proteins (40, 41). Accordingly, we investigated the possibility of direct interaction between two proteins, d-IIA^Glc^ and d-DctD_2_, since both accumulated in the cells under the glucose-rich condition. A β-galactosidase-based bacterial two-hybrid system (BACTH) (Euromedex) was utilized. Two plasmids, pUT18c and pKT25, containing *dctD_2_* and *crr*, respectively, were cotransformed into Escherichia coli. The resultant transformant was spotted on M9-glucose or M9-phosphoenolpyruvate (PEP) agar plates supplemented with 40 μg/mL X-Gal (5-bromo-4-chloro-3-indolyl-β-d-galactopyranoside) (Fig. 5A and C). For comparison, the negative and positive controls, which were E. coli harboring a combination of pUT18c and pKT25 vectors and a combination of pUT18c-*zip* and pKT25-*zip*, respectively, were also spotted on the same agar plates. Blue colonies of the cells carrying pUT18c-*dctD_2_* and pKT25-*crr* formed on the M9-glucose plate, but not on the M9-PEP plate, which suggested that only d-IIA^Glc^ exhibited an affinity for DctD_2_. The measured activities of β-galactosidase produced in M9-glucose and M9-PEP were the same as those of the positive control and negative control, respectively (Fig. 5B and D).

10.1128/mbio.03839-21.3 TABLE S1 Strains and plasmids used in this study. Download Table S1, DOCX file, 0.02 MB. Copyright © 2022 Kang and Lee. 2022 Kang and Lee. https://creativecommons.org/licenses/by/4.0/ This content is distributed under the terms of the Creative Commons Attribution 4.0 International license.

It was assumed that most DctD_2_ proteins in cells grown in glucose, without any dicarboxylic acid, existed as d-DctD_2_, based on a prior finding (42). To identify which form of DctD_2_ participates in binding with d-IIA^Glc^, *in vitro* binding assays were performed using various combinations of the two proteins (Fig. 5E). Native gel electrophoresis analysis revealed a novel band in samples with a combination of DctD_D57Q_ (d-DctD_2_) with d-IIA^Glc^. It indicated the formation of a complex of the two proteins, whose intensities were apparently dependent upon the concentration of each protein. However, a combination of DctD_D57E_ (p-DctD_2_) with d-IIA^Glc^ did not produce any new band, suggesting that p-DctD_2_ was not able to interact with d-IIA^Glc^ (the last lane in Fig. 5E).

### d-DctD_2_ specifically bound to the upstream region of the EPS-cluster if d-IIA^Glc^ was provided.

**FIG 6:**
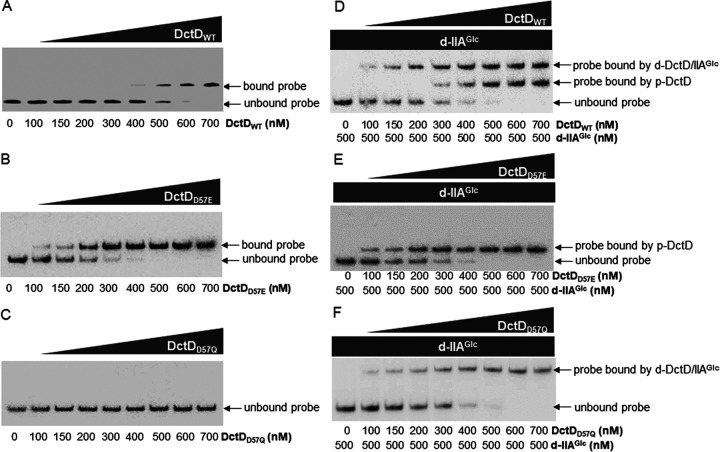
Binding affinities of p-DctD_2_ and d-DctD_2_/d-IIA^Glc^ complex to the regulatory region of the EPS-II cluster. The labeled DNA probe (50 nM), covering −391 to +61 relative to the TIS of the EPS-II gene cluster (16), was incubated with DctD_WT_ (A), DctD_D57E_ (B), and DctD_D57Q_ (C) at concentrations ranging from 100 to 700 nM. To examine the role of d-IIA^Glc^ in DNA binding of DctD, the same mixtures were also added with 500 nM d-IIA^Glc^ (D, E, and F). The reaction mixtures were resolved in 6% native polyacrylamide gels. Lane 1, probe only; and lanes 2 to 9, probe with DctD proteins at a concentration of 100, 150, 200, 300, 400, 500, 600, or 700 nM DctD_WT_. DNA probes bound by p-DctD (A, B, D, and E) or a complex of d-DctD with d-IIA^Glc^ (D and F) are indicated with arrows.

Phosphorylation of bEBPs occurs prior to their binding to DNA (12). In the case of V. vulnificus DctD_2_, which activates the transcription of EPS-II and EPS-III clusters, its phosphorylation state was suggested to result from kinase activity of the cognate sensors for dicarboxylic acids (16). However, in the presence of glucose, transcriptional activation of the EPS-II and EPS-III clusters required the presence of d-IIA^Glc^ and d-DctD_2_ (Fig. 4). Both showed a specific interaction to form a complex (Fig. 5). Thus, we examined whether the complex of d-DctD_2_ and d-IIA^Glc^ would interact with the regulatory region of the target genes by performing an electrophoretic mobility shift assay (EMSA) with three kinds of recombinant DctD_2_: the original, wild-type (WT) DctD_2_ (DctD_WT_), DctD_D57E_ (p-DctD_2_), and DctD_D57Q_ (d-DctD_2_). They were individually added to the reaction mixtures containing 50 nM probe DNA covering the nucleotides from −418 to +62 relative to the transcription initiation site 1 (TIS-1) of the EPS-II gene cluster (Fig. 6). As previously reported, DctD_WT_ bound to the probe in a concentration-dependent manner (16) (Fig. 6A). It was presumed that the DctD_WT_ preparations used in this study contained both phosphorylated and dephosphorylated forms of DctD_2_. Thus, the recombinant DctD_2_ preparations including only one form (either p-DctD_2_ or d-DctD_2_) were added to the same DNA probe. p-DctD_2_ began to bind the probe in the reaction mixture with the lowest concentration of DctD_2_ (e.g., 100 nM), which had approximately 3 times higher affinity than DctD_WT_, and the intensity of the bound probes increased as the added concentrations of p-DctD_2_ increased up to 500 nM (Fig. 6B). However, as expected, d-DctD_2_ did not show any affinity for the same probe up to the highest concentration of DctD used in this study (e.g., 700 nM) (Fig. 6C). These results clearly demonstrate that d-DctD_2_ has no DNA-binding affinity.

The same EMSA was performed in the presence of d-IIA^Glc^. When the DNA probes mixed with various concentrations of DctD_WT_ were treated with 500 nM d-IIA^Glc^, a slowly migrating band emerged in a concentration-dependent manner (Fig. 6D), in addition to a band of the probes bound by p-DctD_2_ (Fig. 6A). We hypothesized that the d-DctD_2_ fractions comprising the DctD_WT_ preparations can interact with d-IIA^Glc^ to form a complex that specifically binds to the probe DNA. To test this hypothesis, p-DctD_2_ and d-DctD_2_ were separately added to the reaction mixtures containing 50 nM DNA probes and 500 nM d-IIA^Glc^. No slowly migrating band was evident in the reaction mixtures containing p-DctD_2_ (Fig. 6E). However, the band indicating the probes bound by the complex of d-DctD_2_ and d-IIA^Glc^ appeared in a d-DctD_2_ concentration-dependent manner up to 500 nM (Fig. 6F). These results demonstrated that d-DctD_2_, which was not able to bind DNA by itself, gained DNA-binding affinity not with its phosphorylation process, but through its binding with d-IIA^Glc^.

### Cellular levels of DctD_2_ and IIA^Glc^ in V. vulnificus cells grown in fumarate, glucose, mannose, or glycerol.

**FIG 7:**
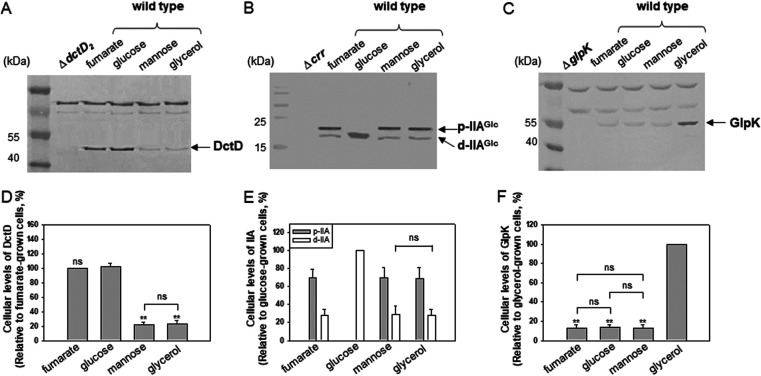
Cellular levels of DctD_2_, IIA^Glc^, and GlpK proteins in V. vulnificus cells grown in fumarate, glucose, mannose, or glycerol. Protein levels of DctD_2_ (A), IIA^Glc^ (B), and GlpK (C) in wild-type V. vulnificus cells, which were freshly grown in AB-fumarate, -glucose, -mannose, or -glycerol medium (up to an OD_595_ of 0.4), were compared by Western blot analysis. For SDS-PAGE, 120, 20, and 50 μg of cell lysates were loaded to detect bands of DctD_2_, IIA^Glc^, and GlpK, respectively. As a negative control for each blot, lysates of the *dctD*, *crr*, or *glpK* deletion mutants were included. The intensities of the corresponding protein bands on each blot were quantified, and their relative amounts (normalized by the intensities of DctD_2_ in cells grown in AB-fumarate [D], IIA^Glc^ in cells grown in AB-glucose [E], and GlpK in cells grown in AB-glycerol [F]) were plotted, with *P* values indicated as follows: **, *P* < 0.001; ns, not significant.

Expression of the transcriptional reporters for *ntrC* and *dctD_2_* genes was highly induced in V. vulnificus cells grown in AB-fumarate and AB-glucose, compared to the cells grown in AB-mannose or AB-glycerol (Fig. 3A). To monitor the cellular levels of DctD_2_, V. vulnificus cells were harvested at the exponential phase (at an optical density at 595 nm [OD_595_] of 0.4) in each growth medium. Western blot analysis using anti-DctD_2_ antibodies revealed that approximately 4.5-times-higher levels of DctD_2_ were present in the cells grown in AB-fumarate and AB-glucose than in AB-mannose and AB-glycerol (Fig. 7A and D). These results were consistent with the expression patterns of the *dctD_2_*-reporter in each growth medium (Fig. 3B). Although the total levels of DctD_2_ were similarly higher in the cells grown in the AB-fumarate and AB-glucose, most DctD_2_ proteins were presumed to be the dephosphorylated form in the AB-glucose-grown cells. Next, the cellular abundance of d-IIA^Glc^ in cells grown in various carbon sources was further monitored. As expected from previous reports in many bacterial species (43), the cells grown in AB-glucose contained only d-IIA^Glc^ (Fig. 7B and E). In contrast, the cells grown in the presence of fumarate, mannose, or glycerol contained both d-IIA^Glc^ and p-IIA^Glc^ at ratios of 1:2.5, 1:2.4, or 1:2.5, respectively. These results support our interpretation of high expression of the EPS clusters in the presence of glucose, in which increased levels of d-IIA^Glc^ would suffice for the complex formation occurring with increased d-DctD_2_, which could then activate transcription of the clusters.

### Increased levels of GlpK in V. vulnificus cells grown with glycerol.

Comparative analyses of the cellular levels of DctD_2_ and IIA^Glc^ revealed similar levels in V. vulnificus cells grown in mannose and glycerol (Fig. 7A and B). Interestingly, expression of the EPS-II and -III clusters (Fig. 2B and C), production of EPS (Fig. 1D), and formation of biofilms (Fig. 1B) were significantly lower in the cells grown in AB-glycerol than in AB-mannose. These observations suggested the presence of a factor inhibiting d-DctD_2_/d-IIA^Glc^ complex-mediated transcription of the EPS clusters, especially in cells grown with glycerol. Since it has been previously reported that the glycerol uptake system, such as GlpFK (44), is highly induced in the presence of glycerol (45), the cellular levels of GlpK were monitored in the cells grown using various carbon sources. Significantly increased levels of GlpK were observed in cells grown in glycerol, which were approximately 7.5 times higher than the levels of GlpK in cells grown in fumarate, glucose, or mannose (Fig. 7C and F).

The direct interaction between GlpK and d-IIA^Glc^ has been described in E. coli (46). This might compete with the interaction between d-DctD_2_ and d-IIA^Glc^. Thus, to further investigate the effect of increased GlpK on the levels of d-IIA^Glc^ that would interact with d-DctD_2_, it was first examined whether V. vulnificus GlpK would interact with IIA^Glc^ (see Fig. S2A in the supplemental material). As shown in E. coli, two proteins of V. vulnificus also exhibited strong binding affinity to form a complex. Next, the possibility of direct interaction of GlpK with DctD_2_ was examined, but no interaction between these two proteins was revealed (Fig. S2B). These results might explain the lowest expression of the EPS gene clusters in V. vulnificus cells grown with glycerol, due to the increased GlpK, which in turn decreased the cellular levels of the free d-IIA^Glc^ available for complex formation with d-DctD_2_.

10.1128/mbio.03839-21.2 FIG S2 Interaction among GlpK, DctD_2_, and d-IIA^Glc^ proteins. (A) Interaction of GlpK with d-IIA^Glc^. To examine the presence of specific interaction between the V. vulnificus IIA^Glc^ and GlpK, as shown in E. coli (46), recombinant d-IIA^Glc^ and GlpK were prepared. Various concentrations of d-IIA^Glc^ and GlpK were mixed, and the reaction mixtures were run in a native gel. Lane 1, d-IIA^Glc^ (3 μM); lanes 2 to 4, GlpK (3 μM) with d-IIA^Glc^ at concentrations of 1.5, 3, and 6 μM; lane 5, GlpK (3 μM); and lanes 6 to 8, d-IIA^Glc^ (3 μM) with GlpK at concentrations of 1.5, 3, and 6 μM. Each protein band and the newly emerged band are indicated with arrows. (B) No interaction of GlpK with DctD_2_. Two recombinant proteins of V. vulnificus, DctD_2_ and GlpK, were purified and mixed in various combinations. The reaction mixtures separated in a native gel were observed if a new band had emerged due to a specific interaction between two proteins. Lanes 1 to 3, DctD_2_ (5 μM) with GlpK at concentrations of 1, 2.5, and 5 μM; lane 4, GlpK (5 μM); lanes 5 to 7, GlpK (5 μM) with DctD_2_ at concentrations of 1, 2.5, and 5 μM; and lane 8, DctD_2_ (5 μM) only. Each protein band is indicated with arrows. Download FIG S2, TIF file, 2.8 MB. Copyright © 2022 Kang and Lee. 2022 Kang and Lee. https://creativecommons.org/licenses/by/4.0/ This content is distributed under the terms of the Creative Commons Attribution 4.0 International license.

### DNA-binding affinity of the d-DctD_2_/d-IIA^Glc^ complex was inhibited by GlpK.

**FIG 8:**
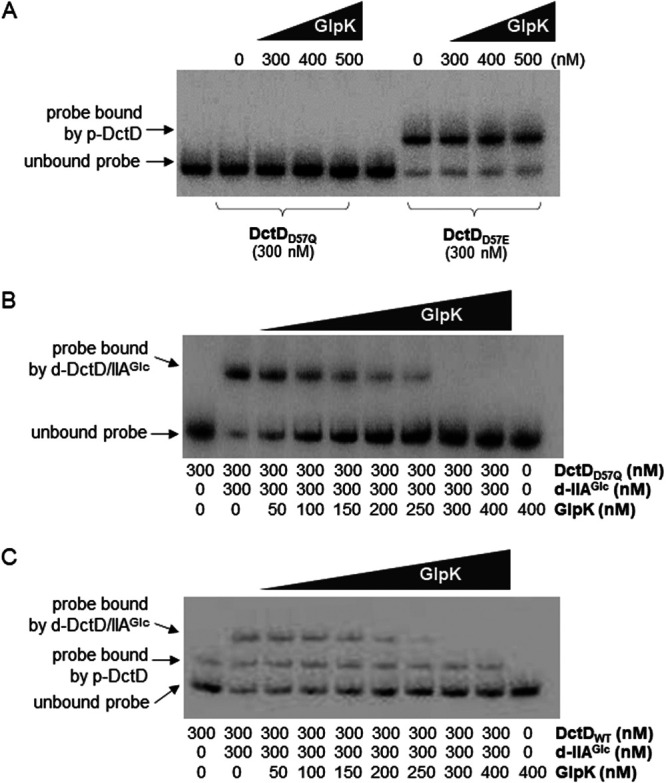
Effect of GlpK on the binding ability of the d-DctD_2_/d-IIA^Glc^ complex to DNA probe. (A) Addition of GlpK to the probe mixed with d-DctD_2_ or p-DctD_2_. Labeled DNA probes (50 nM), used in Fig. 6, were mixed with either 300 nM DctD_D57Q_ (lanes 3 to 5) or 300 nM DctD_D57E_ (lanes 8 to 10). To examine the effect of GlpK on the interaction of DctD_2_ and DNA probe, various concentrations of recombinant GlpK ranging from 300 to 500 nM were added, and then the reaction mixtures were resolved in a 6% native polyacrylamide gel. Lanes 1 and 6, DNA probe only; and lanes 2 and 7, DNA probe with 300 nM DctD_2_. Unbound DNA and the probes bound by p-DctD_2_ are indicated with arrows. (B) Addition of GlpK to the probe mixed with d-DctD and d-IIA^Glc^. Labeled DNA probes (50 nM) mixed with 300 nM DctD_D57Q_ and 300 nM d-IIA^Glc^ were further mixed with various concentrations of recombinant GlpK ranging from 50 to 400 nM (lanes 3 to 9). The reaction mixtures were resolved in a 6% native polyacrylamide gel. Lane 1, DNA probe with DctD_D57Q_ (300 nM); lane 2, DNA probe with DctD_D57Q_ (300 nM) and d-IIA^Glc^ (300 nM); and lane 10, DNA probe with GlpK (400 nM). Unbound DNA and the probes bound by a complex of d-DctD_2_ and d-IIA^Glc^ are indicated with arrows. (C) Addition of GlpK to the probe mixed with DctD_WT_ and d-IIA^Glc^. Labeled DNA probes (50 nM), mixed with 300 nM DctD_WT_ (which included both d-DctD_2_ and p-DctD_2_) and 300 nM d-IIA^Glc^, were further mixed with various concentrations of recombinant GlpK ranging from 50 to 400 nM (lanes 3 to 9). The reaction mixtures were resolved in a 6% native polyacrylamide gel. Lane 1, DNA probe with DctD_WT_ (300 nM); lane 2, DNA probe with DctD_WT_ (300 nM) and d-IIA^Glc^ (300 nM); and lane 10, DNA probe with GlpK (400 nM). DNA probes bound by p-DctD_2_ or a complex of d-DctD_2_ with d-IIA^Glc^ are indicated with arrows.

The observations that d-IIA^Glc^ was able to form complexes with d-DctD_2_ (Fig. 5E) and GlpK (Fig. S2A) prompted a further examination of whether the formation of the d-IIA^Glc^/GlpK complex would interfere with the formation of d-IIA^Glc^/d-DctD_2_ by examining the behavior of the d-IIA^Glc^/d-DctD_2_ complex in response to the target DNA probe in the presence of various concentrations of GlpK. It was first checked if GlpK would affect the DNA-binding affinity of both forms of DctD_2_ in the absence of d-IIA^Glc^, but GlpK did not cause any change in the DNA-binding affinity of both forms of DctD_2_ (Fig. 8A). However, in the presence of added GlpK, the amount of DNA probe bound by the d-IIA^Glc^/d-DctD_2_ complex was apparently decreased in a GlpK concentration-dependent manner, with a coincidental increase in the intensities of the unbound probe (Fig. 8B). The DNA probes bound by the complex completely disappeared in the reaction mixtures containing GlpK at concentrations equal to or greater than those of d-IIA^Glc^ and d-DctD_2_ (≥300 nM). As expected from the specificity of the interaction of GlpK with d-IIA^Glc^, the band intensities of DNA probes bound by p-DctD_2_ were not affected by the addition of GlpK (Fig. 8C). In contrast, the band intensities of DNA probes bound by the d-IIA^Glc^/d-DctD_2_ complex were affected by the addition of GlpK, and the bands eventually disappeared in the presence of higher concentrations of GlpK. The findings from all the experiments using GlpK support the hypothesis that GlpK inhibits the formation of a transcriptionally active form of d-DctD_2_, especially in cells grown under glycerol-enriched conditions.

### Biofilm formation by mutants defective in IIA^Glc^ or GlpK.

**FIG 9:**
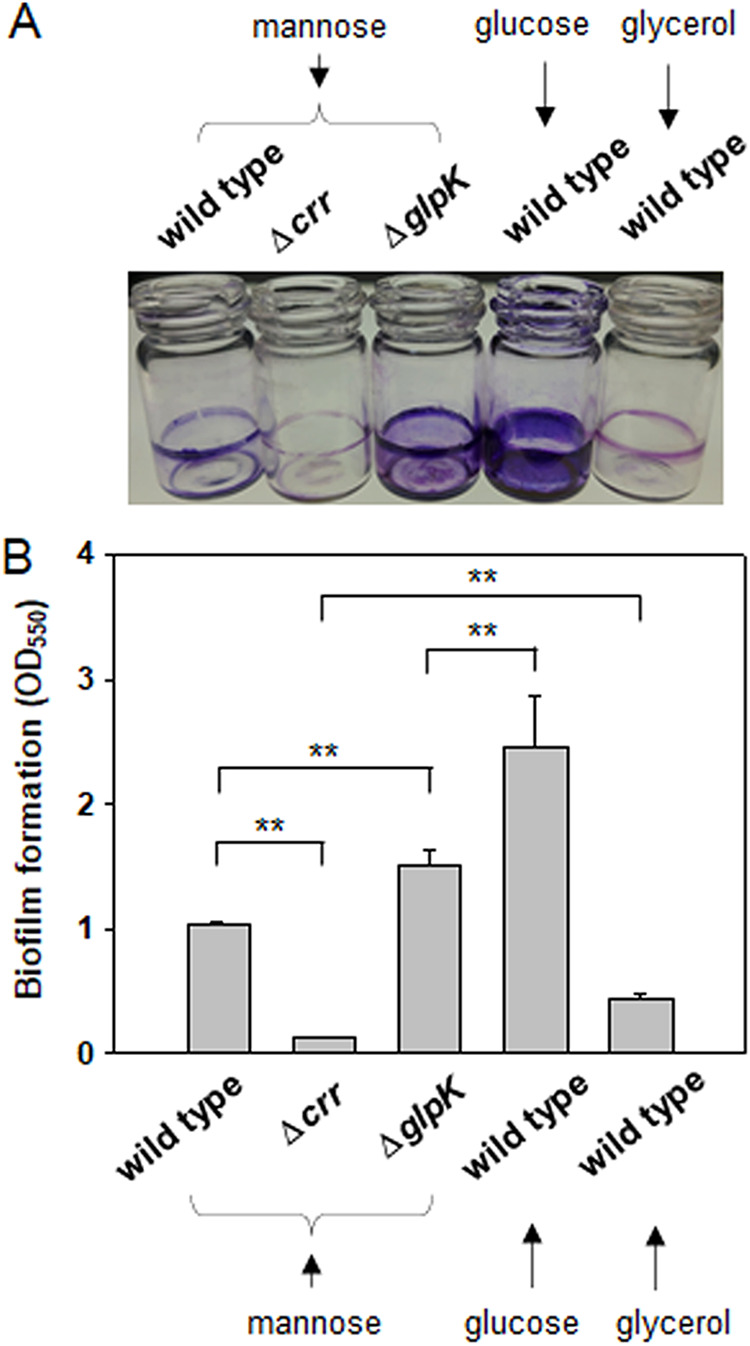
Biofilm formation by Δ*crr* and Δ*glpK* mutant strains of V. vulnificus in the presence of mannose. The wild-type and Δ*crr* and Δ*glpK* mutant strains were statically incubated for 48 h in AB medium supplemented with mannose, and the resultant biofilms on the borosilicate tubes were estimated by staining with crystal violet (A). For comparison, biofilms formed by the wild type in AB medium supplemented with glucose or glycerol were included. The associated dyes were dissolved and measured by spectrophotometry at 550 nm (B). The *P* values are indicated above corresponding horizontal lines: **, *P* < 0.001; *, 0.001 ≤ *P* < 0.01; ns, not significant.

To verify the *in vivo* effect derived from the competitive relationship between d-DctD_2_ and GlpK in forming a complex with d-IIA^Glc^ (Fig. 8), one of the V. vulnificus phenotypes related to the degree of expression of the EPS gene clusters was examined. Since maturation of biofilms is tightly dependent upon EPS production (28, 31), the extents of mature biofilms developed by V. vulnificus wild-type and Δ*crr* and Δ*glpK* mutant strains were compared in AB-mannose (i.e., in the absence of glucose and glycerol) (Fig. 9A). Biofilm formation by the wild type in AB-mannose was approximately 2.1 times lower than that in AB-glucose and 3.4 times higher than that in AB-glycerol (Fig. 9B). These values were intermediate among the degrees of biofilm formation in AB-glucose and AB-glycerol media (as shown in Fig. 1A and B), which could be supported by the expression results of the EPS-II and EPS-III clusters in AB-mannose (as shown in Fig. 2B and C). Deletion of the *crr* gene encoding IIA^Glc^ resulted in a basal level of biofilm formation, which was slightly but significantly lower than the level of biofilm formation by the wild type in glycerol, due to the absence of a factor transforming the transcriptionally inactive d-DctD_2_ into the transcriptionally active d-DctD_2_/d-IIA^Glc^ complex. In contrast, biofilms formed by the Δ*glpK* mutant in AB-mannose were significantly increased compared to those by the wild type grown in the same medium, possibly because of the increased fraction of free d-IIA^Glc^ capable of forming a complex with d-DctD_2_ in the Δ*glpK* mutant. However, these levels were significantly lower than biofilms formed by the wild type grown in AB-glucose, in which both d-DctD_2_ and d-IIA^Glc^ were significantly increased (Fig. 7A and B).

## DISCUSSION

**FIG 10:**
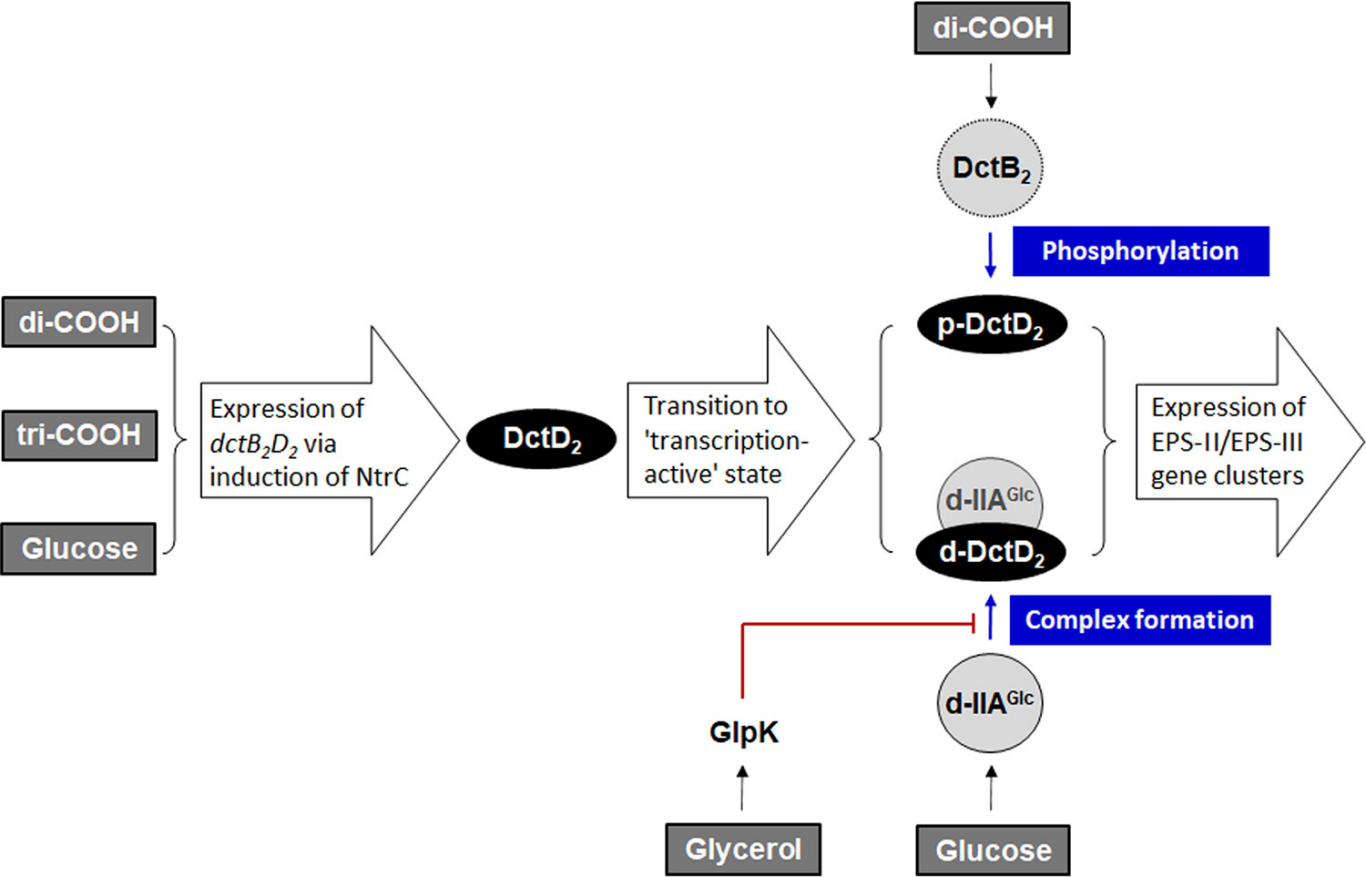
DctD_2_-directed regulatory pathways for expression of two gene clusters for EPS-II/III biosynthesis in response to various carbon sources. Production of EPS, which is essential for V. vulnificus to mature biofilms, is controlled at the level of transcription of the gene clusters for EPS biosynthesis (EPS clusters). Thus, the cellular abundance of transcriptionally active forms of DctD_2_ responsible for regulating the EPS-II and EPS-III clusters (16) determines the degrees of EPS production and biofilm maturation. Transcription of *dctD_2_* is activated by NtrC, whose expression is induced under conditions with TCA intermediates (i.e., di- and tricarboxylic acids) (16) and glucose. In the presence of dicarboxylic acids, most DctD_2_ is present in the transcriptionally active state as a phosphorylated form (p-DctD_2_) by its cognate sensor kinase DctB_2_ (42). The transcriptionally inactive, dephosphorylated form of DctD_2_ (d-DctD_2_) is capable of activating the transcription of EPS gene clusters in cells grown with glucose, in which d-DctD_2_ forms a complex with d-IIA^Glc^. This d-DctD_2_-mediated transcription of the EPS-gene clusters is reduced in the presence of glycerol, due to a competitive inhibition of GlpK against the formation of the d-DctD_2_/d-IIA^Glc^ complex.

It has been considered that the transcriptionally active form of enhancer-binding response regulators belonging to TCS is in the phosphorylated state, which is achieved by their cognate sensor kinases under specific conditions (12). In addition to the signal transduction mechanism via phospho-relay, this study further identified a novel regulatory pathway to activate transcription, which is mediated by the dephosphorylated form of a response regulator without involvement of its cognate sensor kinase, as schematically summarized in Fig. 10. One of the well-known response regulators, DctD_2_, is present in the phosphorylated and active form when bacterial cells are grown in the presence of dicarboxylic acids (16). However, upon growth in the presence of glucose, the dephosphorylated form of DctD_2_ is able to efficiently activate the transcription of EPS gene clusters in a dicarboxylic acid-independent manner (Fig. 2 and 4). This glucose-dependent transition of d-DctD_2_ from a transcriptionally inactive to transcriptionally active state, evidenced by the acquisition of DNA-binding ability (as shown in Fig. 6), necessitates the involvement of another cellular factor indicating the availability of glucose in the ambient environment. Components of the glucose PTS are mostly present in the dephosphorylated states in bacterial cells growing in the presence of glucose (47) (Fig. 7B), and d-IIA^Glc^ has been shown to specifically interact with d-DctD_2_ (Fig. 5). Since p-DctD_2_ would be in the multimeric states in order to bind to DNA, as shown in other members of the NtrC family (12), it was further speculated that a multimeric conformation of the d-IIA^Glc^/d-DctD_2_ complex might be involved in direct binding to DNA. This speculation could be supported by the observation that a DNA probe bound by the d-IIA^Glc^/d-DctD_2_ complex showed more retarded mobility than the same DNA probe bound by p-DctD_2_ (Fig. 6D).

In addition to the role of the glucose PTS in translocating glucose molecules into the cytoplasm in a phosphorylated form (glucose-6-phosphate), its components are versatile proteins that interact with diverse proteins and regulate their cellular functions (41). In the case of IIA^Glc^ relaying the phosphate group from HPr to IIBC^Glc^, it has been further reported that IIA^Glc^ specifically interacts with enzymes (Cya, FrsA, AraG, MalK, and GlpK), non-PTS transporters (LacY, GalP, MelB, and RafB), and chemotactic signal transducers (CheW) in several bacterial species (48–50). Besides these proteins targeted by IIA^Glc^, some transcription factors are affected by other components of the glucose PTS. The membrane-bound IIB^Glc^ binds to a transcription factor, Mlc, and then sequesters it from its transcription regulation of the target genes in E. coli (51), and HPr makes MtlR an active transcription factor by direct phosphorylation in Bacillus subtilis (52). The present study demonstrates that IIA^Glc^ can form a complex with the inactive DctD_2_ and convert it to an active DctD_2_ having the DNA-binding affinity. This is the first evidence that the interaction of IIA^Glc^ with a transcription factor results in an effect mimicking the phosphorylated form of a response regulator.

As mentioned above, d-IIA^Glc^ was able to form a complex with glycerol kinase, GlpK (Fig. S2A). This led us to speculate that GlpK plays a role in the expression of genes regulated by the d-DctD_2_/d-IIA^Glc^ complex, especially under conditions that enrich the cellular abundance of GlpK. Cellular levels of GlpK were highly induced in cells grown in AB-glycerol medium (Fig. 7C), and GlpK might outcompete d-DctD_2_ in forming the complex with d-IIA^Glc^. As a result, the potential to form the d-DctD_2_/d-IIA^Glc^ complex was presumed to be lowered in the cells growing with glycerol compared to the cells growing with mannose (Fig. 7B). This competitive interaction between d-DctD_2_ and GlpK was evidenced *in vitro* by EMSA (Fig. 8). Furthermore, when comparing the intensities of the probes bound by various concentrations of the d-DctD_2_/d-IIA^Glc^ complex (Fig. 6F) with those in the presence of various amounts of added GlpK (Fig. 8B), the binding affinity of GlpK to d-IIA^Glc^ appeared to be slightly higher than that of d-DctD_2_ to d-IIA^Glc^. The *in vivo* effect caused by the increased levels of GlpK was confirmed by observing the biofilm phenotype of the Δ*glpK* mutant. This mutant exhibited the ability to form significantly increased biofilms in AB-mannose medium compared to the wild type in the same growth medium (Fig. 9).

Both the production of EPS and the formation of mature biofilms were greatly increased when cells were grown in the presence of glucose (Fig. 1). These processes were accomplished by sequential induction of *ntrC* and *dctD_2_* gene expression (Fig. 3), accumulation of d-IIA^Glc^ (Fig. 7B), complex formation of d-DctD_2_ with d-IIA^Glc^ (Fig. 5), and transcriptional activation of the gene clusters for EPS biosynthesis (Fig. 2 and 4). NtrC is the main transcription factor activating the *dctB_2_D_2_* operon in V. vulnificus (16). Induction of NtrBC via sensing the cellular fluctuations of glutamate (Glu) and glutamine (Gln) as a carbon/nitrogen ratio has been documented. In the cells growing with the tricarboxylic acid (TCA) intermediates (i.e., tri- and dicarboxylic acids), the activities of α-ketoglutarate (α-KG) dehydrogenase, catalyzing conversion of α-KG to succinyl coenzyme A (succinyl-CoA), and succinate dehydrogenase, catalyzing conversion of succinate to fumarate, are inhibited. Then, the increased cellular levels of α-KG result in increased levels of Glu, which activates NtrBC (53–55). Under glucose-rich conditions, the cellular levels of cAMP are minimal, resulting in relief of the repression of the *ntr* operon by the cAMP-cAMP receptor protein (CRP) complex (56). Therefore, under conditions that result in increased levels of NtrC (e.g., TCA intermediates or glucose), the expression of DctD_2_ is highly induced. The induced cellular level of DctD_2_ is not the only requirement for maximal activation of the EPS gene clusters’ transcription. DctD_2_ needs to be converted to the transcriptionally active state: the p-DctD_2_ is formed in the presence of dicarboxylic acids among the TCA intermediates (16), and the d-DctD_2_/d-IIA^Glc^ complex is formed in the presence of glucose (Fig. 5A).

This series of regulations of EPS production may be disrupted by glycerol. It is conceivable that the addition of glycerol might reduce the gluconeogenesis process producing the key sugars of EPS and/or the other processes catalyzing carbon storage in the form of carbon-rich polymers. As evidenced by the highly induced expression of GlpK by glycerol (Fig. 7C) and its antagonistic effect on the formation of the d-DctD_2_/d-IIA^Glc^ complex (Fig. 8), GlpK could play an important role in guaranteeing the reduced expression of EPS gene clusters, even in cells with some amounts of d-DctD_2_ and d-IIA^Glc^. DctD_2_ in V. vulnificus cells growing in AB-glycerol should be in the dephosphorylated state, but their IIA^Glc^ proteins are present in both forms, p-IIA^Glc^ and d-IIA^Glc^ (Fig. 7B). Thus, the cellular levels of d-IIA^Glc^ available to form a complex of d-DctD_2_ with d-IIA^Glc^ should be limited in the cells growing in AB-glycerol medium, due to the increased levels of GlpK. As a result, these cells showed a significantly decreased ability to produce EPS and form biofilms compared to the cells growing in AB-mannose medium (Fig. 1 and 2). Similarly, other non-PTSs might have the same inhibitory effect. The inhibitory effect of GlpK, with higher affinity than d-DctD_2_, might suggest a role similar to that of other non-PTS transporters, guaranteeing the reduced expression of EPS gene clusters. In addition to the role of glycerol in the inhibition of d-DctD_2_/d-IIA^Glc^ complex formation via the involvement of GlpK, the other carbon sources could have the same effect as glycerol, if their kinases are able to interact with d-IIA^Glc^: for example, maltose and its kinase, MalK (57), or arabinose and its kinase, AraG (58).

Production of EPS and formation of mature biofilms are important for increased colonization and survival of V. vulnificus in various environments, including its hosts. Since EPS biosynthesis is regulated at the level of transcription in V. vulnificus (28), the fine-tuned regulation of EPS biosynthesis is required to sense the ambient environmental parameters, such as the types of carbon sources, and to initiate and prepare a biofilm lifestyle. Transcription initiation activated by bEBP, such as DctD, occurs in RpoN-dependent TIS (12). As previously reported (16), the gene clusters for EPS-II and EPS-III have both RpoN-dependent TIS (TIS-1) and RpoD-dependent TIS (TIS-2). The observations of the effects of carbon sources on the DctD_2_-mediated activation shown in this study are the results of the induction at TIS-1 of each cluster. The transcription of the EPS-II gene cluster (also called the *brp* operon) can be induced at its TIS-2 by the c-di-GMP-responding transcription factors BrpR and BrpT (30, 59).

This study demonstrates that the foodborne pathogen V. vulnificus utilizes both dicarboxylic acids and glucose as the specific signals to turn on the transcription of the EPS gene clusters and the sources to supply the monomeric constituents or their precursors for EPS polymerization, but efficiently adopts the differential pathways using a common response regulator, DctD_2_, in the form of p-DctD_2_ or d-DctD_2_/d-IIA^Glc^ complex. These findings reveal a novel regulatory mechanism for EPS biosynthesis in glucose-enriched environments, which foodborne pathogens might encounter upon entry into the gastrointestinal tracts of hosts. The pathway mediated by the d-DctD_2_/d-IIA^Glc^ complex is further delicately regulated by other carbon sources, such as glycerol, via the competitive action of their kinases inhibiting the formation of the d-DctD_2_/d-IIA^Glc^ complex.

## MATERIALS AND METHODS

### Bacterial strains and culture conditions.

All the strains and plasmids used in this study are separately listed in Table S1 in the supplemental material. The E. coli strains used for plasmid DNA preparation and for conjugational transfer were grown at 37°C in Luria-Bertani (LB) medium. V. vulnificus strains were grown at 30°C in AB medium (300 mM NaCl, 50 mM MgSO_4_, 0.2% vitamin-free Casamino Acids, 10 mM potassium phosphate or 100 mM sodium phosphate, 1 mM l-arginine, pH 7.5) (60) supplemented with various carbon sources: fumarate (50.0 mM), glucose (33.4 mM), mannose (33.4 mM), or glycerol (66.7 mM). Antibiotics were added to LB or AB media at the following concentrations: for E. coli, ampicillin at 100 μg/mL, chloramphenicol at 20 μg/mL, kanamycin at 50 μg/mL, and tetracycline at 15 μg/mL, and for V. vulnificus, chloramphenicol at 2 μg/mL and tetracycline at 3 μg/mL.

### Biofilm formation assay.

Wild-type and mutant strains of V. vulnificus were freshly inoculated to the AB media supplemented with various carbon sources in borosilicate tubes and statically incubated at 30°C. At 48 h, the planktonic phases were removed from the tubes, and their cell densities were measured by spectrometric reading at OD_595_ to check the appropriate growth of cells on the specific carbon sources. Biofilms formed on tubes were washed twice with phosphate-buffered saline (PBS: 100 mM NaCl, 20 mM sodium phosphate [pH 7.5]) (61), and then the remaining biofilms were stained with 1.0% crystal violet. After briefly washing out the dyes unassociated with biofilms in the tubes with distilled water (DW), the biofilm-associated dyes were eluted in ethanol. The resultant elution was diluted, if necessary, and subjected to spectrometry to estimate OD_550_ (62).

### Extraction and analysis of EPS.

V. vulnificus cells were grown on AB agar plates supplemented with various carbon sources for 48 h, and then the produced EPSs were extracted and quantified, as previously described (28, 63). The fractions of loosely associated extracellular matrix were eluted in PBS and sequentially treated with a mixture of RNase A (50 μg/mL), DNase I (50 μg/mL), and MgCl_2_ (10 mM) for 12 h and then with proteinase K (200 μg/mL) for 8 h. The fractions of polysaccharides remaining in the reaction mixture were treated with phenol-chloroform (1:1 [vol/vol]), and the resultant extracts were treated with sodium acetate (300 mM) to precipitate polysaccharides in the presence of 2.5× volumes of ethanol. The precipitates were washed with 70% ethanol and then resuspended in appropriate volumes of DW based upon the bacterial biomasses used for the EPS extraction: i.e., 100 μL of DW per OD_595_ of 50. Aliquots of EPS fractions were run on a 5% stacking polyacrylamide gel, as described previously (28), and visualized with Stains-All (Sigma). The carbohydrate contents in each EPS fraction were estimated by the phenol-sulfuric acid method, using glucose as a carbohydrate standard (64), to present the concentrations of EPS as the glucose equivalents (Glc-eq.).

### Measurement of transcriptional reporter plasmids fused with the luciferase genes.

The *luxAB*-based transcription reporters, previously described (16, 28), were mobilized to various strains of V. vulnificus. The intensities of the light produced by the cells grown in the presence of specific carbon sources were measured with a luminometer (TD-20/20 luminometer; Turners Designs), after the bacterial culture aliquots were mixed with a luciferase substrate (e.g., *n*-decyl aldehyde [0.006%]). Specific bioluminescence was presented by normalizing the relative light units (RLU) with respect to cell mass (OD_595_), as described previously (65).

### Construction of mutant strains of V. vulnificus.

For construction of the Δ*glpK* mutant, a suicide vector, pDM4 (66), was ligated with a DNA fragment containing the deleted ORFs of *glpK*, which was produced by using two sets of primers: (i) GlpK-upF and GlpK-upR and (ii) GlpK-downF and GlpK-downR (see Table S2 in the supplemental material). pDM4-Δ*glpK* was transformed into E. coli SM10 λ*pir*, and the resultant transformant was conjugated with V. vulnificus. The exconjugants were selected on the appropriate agar plate (i.e., thiosulfate citrate bile sucrose medium supplemented with 3 μg/mL chloramphenicol). Then, an isolated colony with the characteristics indicating a double homologous recombination event (67) was examined by PCR using primers GlpK-upF and GlpK-downR (Table S2) to confirm the mutation in its *glpK* locus. Mutation in Δ*glpK* was further examined via complementation of the mutant with pRK415 containing the intact *glpK* amplified using primers GlpFK-comF and GlpFK-comR (Table S2).

10.1128/mbio.03839-21.4 TABLE S2 Oligonucleotides used in this study. Download Table S2, DOCX file, 0.02 MB. Copyright © 2022 Kang and Lee. 2022 Kang and Lee. https://creativecommons.org/licenses/by/4.0/ This content is distributed under the terms of the Creative Commons Attribution 4.0 International license.

For construction of the mutants whose *dctD_2_* and/or *crr* genes were site-directedly mutagenized, various DNA fragments encompassing these ORFs were prepared by the overlap extension method (68), using appropriate sets of primers (Table S2). For convenience in isolating the mutant among the conjugation mixtures, modified nucleotide sequences for a restriction enzyme were artificially generated in the vicinity of the mutation site without alteration of the original amino acid sequences. For example, the DNA fragments of *dctD*_D57E_ and *dctD*_D57Q_ were produced using the internal primer sets D57E_AgeI-F and D57E_AgeI-R and D57Q_AgeI-F and D57Q_AgeI-R, respectively, including the substituted nucleotide sequences for the altered 57th codon and the restriction site of AgeI. Similarly, *crr*_H75Q_ was produced using the internal primer set H75Q_SacI-F and H75Q_SacI-R. Site-directed mutagenized DNA fragments were then cloned to pDM4 to produce pDM4*-dctD*_D57E_, pDM4*-dctD*_D57Q_, and pDM4-*crr*_H75Q_, which were transferred to V. vulnificus via conjugation and eventually exchanged with the original ORFs in the V. vulnificus genomes, as described above. Mutations in the target genes were examined by digestion of amplified DNA with the appropriate restriction enzymes (AgeI for *dctD*_D57E_ and *dctD*_D57Q_ and SacI for *crr*_H75Q_).

### Bacterial two-hybrid assay.

Two plasmids, pUT18c and pKT25, provided in the bacterial two-hybrid (BACTH) system (Euromedex) were inserted with *dctD_2_* and *crr*, respectively, and cotransformed into E. coli BTH101. The resultant transformant was spotted on the 5 mM glucose- or 10 mM PEP-supplemented M9–X-Gal (40 μg/mL) agar plate containing ampicillin (100 μg/mL), kanamycin (50 μg/mL), and IPTG (isopropyl-β-d-thiogalactopyranoside; 1 mM). The colony color that developed due to the produced β-galactosidase activity was compared with those of the positive control (E. coli BTH101 carrying pUT18C-*zip*/pKT25-*zip*) and the negative control (E. coli BTH101 carrying containing pUT18c/pKT25). To quantify the degrees of protein-protein interactions in the cells grown in M9 broth with 5 mM glucose or 10 mM PEP, β-galactosidase assays were performed. Harvested cells were resuspended in Z buffer (60 mM Na_2_HPO_4_, 40 mM NaH_2_PO_4_, 10 mM KCl, 1 mM MgSO_4_, and 50 mM mercaptoethanol) (69) and treated with 0.1% SDS and chloroform to prepare crude cell lysates. Appropriate amounts of lysates of cells that were determined by OD_595_ were incubated with ONPG (*o*-nitrophenol-β-galactoside) at a concentration of 0.67 mg/mL, the reactions were stopped by addition of Na_2_CO_3_ solution, and their OD_420_ values were measured. β-Galactosidase activity was presented as follows: 1 U = (OD_420_ × 1,000)/(time × cell culture vol × OD_595_) (69).

### Cloning and purification of recombinant proteins.

For preparation of recombinant GlpK (rGlpK), a 1,546-bp DNA fragment containing the complete *glpK* gene was produced using GlpK-F and GlpK-R, the ends of which contained BamHI and HindIII sites, respectively (Table S2). Utilizing these restriction sites, *glpK* DNA was cloned into pQE30 (Qiagen) and then transformed into E. coli JM109 (Promega). For preparation of three kinds of recombinant DctD_2_ (rDctD_2_)—e.g., the original DctD_2_ (DctD_WT_), p-DctD_2_ (DctD_D57E_), and d-DctD_2_ (DctD_D57Q_)—the plasmids pQE30-*dctD_2_*, pQE-*dctD*_D57E_, and pQE30-*dctD*_D57Q_ were expressed in E. coli JM109 in the presence of 1 mM IPTG. Each recombinant protein was purified using a Ni^+^-nitrilotriacetic acid (NTA) affinity column (Bio-Rad).

### *In vitro* protein-protein interaction assay.

The mutant rDctD_2_ (DctD_D57Q_ and DctD_D57E_) and rGlpK were mixed with the recombinant d-IIA^Glc^ in 20 μL of an assay solution (50 mM Tris-HCl [pH 8.0], 20 mM KCl, 50 mM MgCl_2_, and 100 mM NaCl). Reaction mixtures were composed of various combinations of DctD_D57Q_ and d-IIA^Glc^, whose concentrations of each protein ranged from 0.04 μM to 5.0 μM. After 30 min of incubation at 30°C, the mixtures were combined with a loading buffer (0.01% bromophenol blue, 0.5 M Tris-HCl [pH 6.8], 50% glycerol) (70) and then subjected to nondenaturing gel electrophoresis using a gel made of 12% native polyacrylamide and a running buffer including 40 mM Tris-glycine (pH 8.3). After electrophoresis, the gel was stained with Coomassie brilliant blue to observe a newly emerged band in addition to the bands presenting each recombinant protein.

### Electrophoretic mobility shift assay.

Gel shift assays were performed with the DNA probe (480 bp) covering the regulatory region of the EPS-II cluster, which was amplified using the primer sets EPSII-F and EPSII-R, as previously described (16). Produced DNA fragment was labeled with [γ-^32^P]ATP using T4 polynucleotide kinase (TaKaRa), and the resultant labeled probe (approximately 50 nM) was incubated in a reaction buffer (50 mM Tris-HCl [pH 8.0], 20 mM KCl, 50 mM MgCl_2_, and 100 mM NaCl) with various concentrations of rDctD_2_ (e.g., DctD_WT_, DctD_D57E_, and DctD_D57Q_) in the absence and presence of d-IIA^Glc^ and GlpK. The reaction mixtures were resolved in 6% native polyacrylamide gels, and the unbound probe and the probes bound by rDctD_2_ or DctD_2_/d-IIA^Glc^ were visualized and analyzed using Personal Molecular Imager FX and Quantity One software (Bio-Rad).

### Western blotting.

Cell lysates of wild-type and mutant strains of V. vulnificus were prepared in Tris-buffered saline with Tween 20 (TBST: 150 mM NaCl, 50 mM Tris-HCl, and 0.1% Tween 20) and appropriate amounts of protein extracts (e.g., 120 μg for DctD_2_, 20 μg for IIA^Glc^, and 50 μg for GlpK Western blots) were used for SDS-PAGE. Blotted membranes were blocked with 5% skim milk–TBST, incubated with the polyclonal antibodies raised against each recombinant protein, and then treated with alkaline phosphatase (AP)-conjugated goat anti-mouse IgG (Jackson ImmunoResearch Laboratories, Inc.) for anti-DctD_2_ antibodies or AP-conjugated goat anti-rat IgG (Jackson ImmunoResearch Laboratories, Inc.) for anti-IIA^Glc^ and anti-GlpK antibodies. Using a nitroblue tetrazolium and 5-bromo-4-chloro-3-indolyl phosphate system (Promega), the immunoreactive bands were visualized, and their relative intensities were quantified using a densitometer (Bio-Rad Gel Doc 2000 system).

### Statistical analyses.

Results are expressed as means ± standard deviations of data from at least three independent experiments. Statistical analysis was performed using Student's *t* test (Systat Program, SigmaPlot version 9; Systat Software, Inc.). *P* values are represented by asterisks: *,  0.001 ≤ *P* < 0.01; **, *P* < 0.001.
