# Supplementary figures and images for: Transition of Dephospho-DctD to the Transcriptionally Active State via Interaction with Dephospho-IIAGlc
Source: mBio. 2022 Mar 21;13(2):e03839-21. doi: 10.1128/mbio.03839-21 (PMC9040800; doi:10.1128/mbio.03839-21)

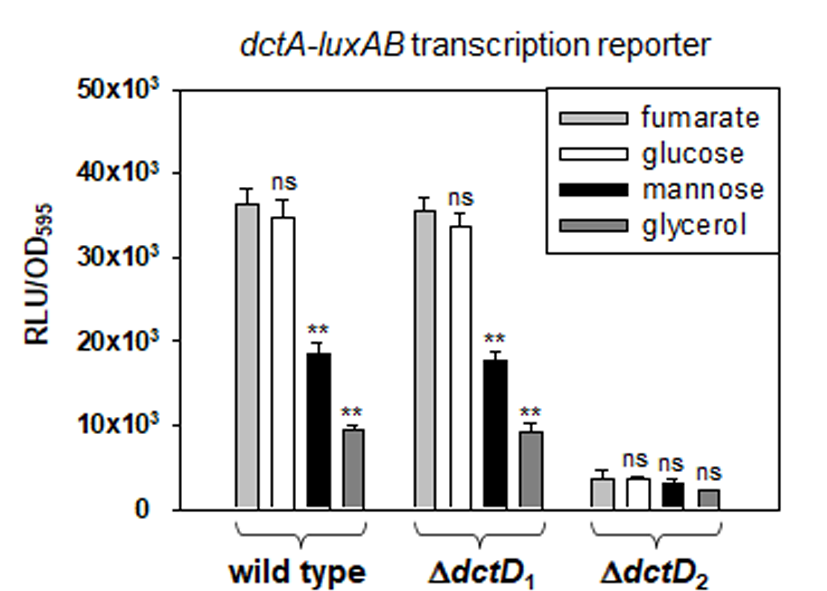

Supplement: FIG S1 [file mbio.03839-21-sf001.tif]

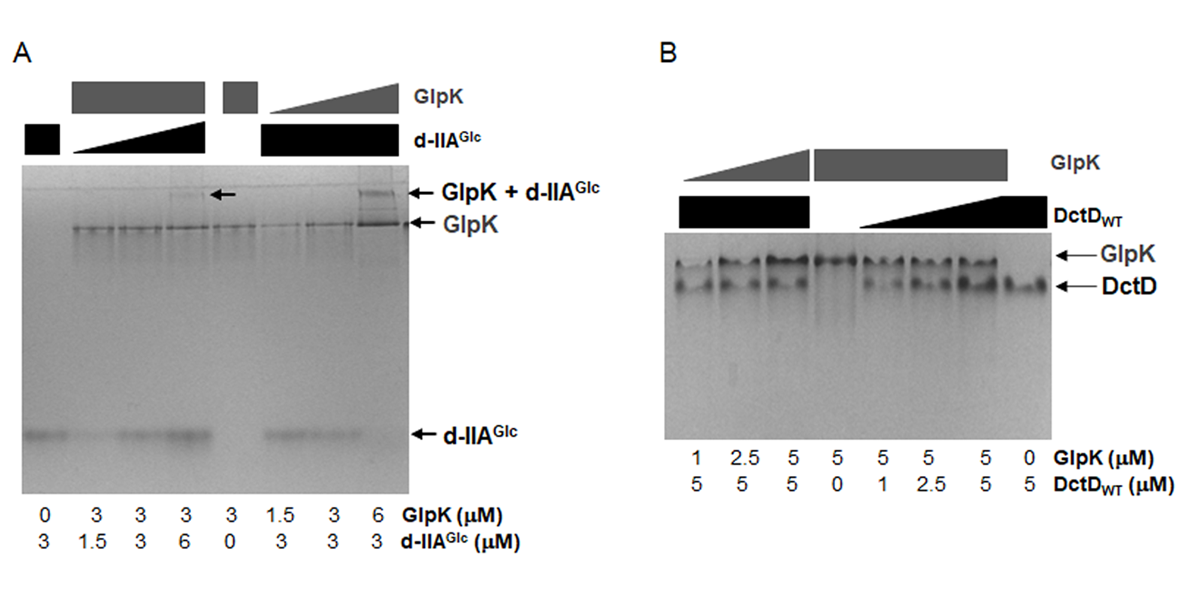

Supplement: FIG S2 [file mbio.03839-21-sf002.tif]
