# Supplementary material for: Transition of Dephospho-DctD to the Transcriptionally Active State via Interaction with Dephospho-IIAGlc
Source: mBio. 2022 Mar 21;13(2):e03839-21. doi: 10.1128/mbio.03839-21 (PMC9040800; doi:10.1128/mbio.03839-21)
Supplement: TABLE S1 [file mbio.03839-21-st001.docx]

**Table S1. Strains and plasmids used in this study**

**Strain/Plasmid Genotype Reference**

***V. vulnificus***

MO6-24/O Clinical isolate (1)

Δ*dctD_1_*  MO6-24/O, Δ*dctD_1_*  (2)

Δ*dctD_2_* MO6-24/O, Δ*dctD_2_* (2)

*dctD*_D57E_ MO6-24/O, *dctD_2_* with 57th amino acid substituted This study

*dctD*_D57Q_ MO6-24/O, *dctD_2_* with 57th amino acid substituted This study

Δ*crr* MO6-24/O, Δ*crr* (3)

*crr*_H75Q_ MO6-24/O, *crr* with 75th amino acid substituted This study

Δ*crr/dctD_2_* MO6-24/O, Δ*crr* and Δ*dctD_2_* This study

Δ*glpK* MO6-24/O, Δ*glpK* This study

***E. coli***

DH5α f80d*lacZ* DM15 *recA1 endA1 gyrA96 relA1* *thi-1* Laboratory collection

*hsdR17*(r_k_^-^ m_k_^-^) *supE44 deoR* *Δ*(*lacZYA-argF*) *U169*

SM10λpir *thi-1 thr leu tonA lacY supE recA*:: (4)

Rp4-2-Tc::Mu*λpir*, OriT of RP4, Km^R^

JM109 *endA1 recA1 gyrA96 thi-1* *hsdR17*(r_k_^-^ m_k_^-^) relA1 Promega

*supE44* (*lac-proAB*)[F’*traD3 6proAB* *lacI*^q^*Z* M15]

BTH101 F- *cya-99 araD139 galE15 galK16 rpsL1* Euromedex

*(Str^r^) hsdR2 mcrA1 mcrB1*

**Plasmids**

pRK415 IncP *ori*, broad-host-range vector *oriT* of RP4, Tc^R^ (5)

pRK415-*glpFK* pRK415 with *V. vulnificus glpFK* ORF This study

pHK0011 pRK415 with promoterless *luxAB*, Tc^R^  (6)

pCB010 *glnA-ntrB-ntrC*::*luxAB* transcriptional fusion in pHK0011 (7)

pCB011 EPS-I cluster::*luxAB* transcriptional fusion in pHK0011 (7)

pCB012 EPS-II cluster::*luxAB* transcriptional fusion in pHK0011 (7)

pCB013 EPS-III cluster::*luxAB* transcriptional fusion in pHK0011 (7)

pHK0011-*dctA* *dctA*::*luxAB* transcriptional fusion in pHK0011 (2)

pHK0011-*dctB_2_D*_2_ *dctB_2_D_2_*::*luxAB* transcriptional fusion in pHK0011 (2)

pBlunt-TOPO Blunt end cloning vector, Km^R^, Ap^R^ MGmed

pTOP-*dctD*_D57E_ pBlunt-TOPO carrying 1,356-bp *dctD_D57E_* This study

pTOP-*dctD*_D57Q_ pBlunt-TOPO carrying 1,356-bp *dctD_D57Q_* This study

pTOP-*crr*_H75Q_ pBlunt-TOPO carrying 522-bp *crr_H75Q_* This study

pDM4 Suicide vector; *ori*R6K, Cm^R^ (8)

pDM4-*dctD_2_*  pDM4 containing ApaI-SacI fragment of Δ*dctD_2_*  (2)

pDM4-*dctD*_D57E_ pDM4 containing ApaI-SacI fragment of pTOP-*dctD_D57E_* This study

pDM4-*dctD*_D57Q_ pDM4 containing ApaI-SacI fragment of pTOP-*dctD_D57Q_* This study

pDM4-*crr*_H75Q_ pDM4 containing ApaI-XbaI fragment of pTOP-*crr_H75Q_* This study

pBlueScriptSKII(+) Cloning vector; Ap^R^ , *lac* promoter, f1, ColE1 Stratagene

pGlpKup pBlueScript SKⅡ(+) with 750-bp upstream region This study

of *glpK*

pGlpKup/down pBlueScript SKⅡ(+) with 601-bp upstream region and This study

696-bp downstream region of *glpK*

pDM4-*glpK*  pDM4 containing XhoI-XbaI fragment of pGlpKup/down This study

pTOP-EPS-II pBlunt-TOPO carrying 480-bp upstream EPS-II cluster This study

pQE30 Expression vector, Ap^R^  Qiagen

pQE30-*dctD_2_* pQE30 containing 1,356-bp *V. vulnificus dctD_2_* (2)

pQE30-*dctD*_D57E_ pQE30 containing 1,356-bp *dctD_D57E_* ORF (2)

pQE30-*dctD*_D57Q_ pQE30 containing 1,356-bp *dctD_D57Q_* ORF This study

pQE30-*crr* pQE30 containing 522-bp *crr* ORF (3)

pQE30-*glpK* pQE30 containing 1,546-bp *glpK* ORF This study

pUT18c BACTH plasmid for T18 fragment of adenylate cyclase Euromedex

(Ap^R^)

pUT18c-*dctD* pUT18c, T18-*dctD_2_* fusion This study

pKT25 BACTH plasmid for T25 fragment of adenylate cyclase Euromedex

(Km^R^)

pKT25-*crr* pKT25, T25-*crr* fusion This study

**References**

1. Wright AC, Simpson LM, Oliver JD, Morris JG Jr. 1990. Phenotypic evaluation of capsular transposon mutants of *Vibrio vulnificus.* Infect Immun 58:1769-1773.

2. Kang S, Park H, Lee KJ, Lee KH. 2021. Transcription activation of two clusters for exopolysaccharide biosynthesis by phosphorylated DctD in *Vibrio vulnificus*. Environ Microbiol 23:5364-5377.

3. Lee KJ, Jeong CS, An YJ, Lee HJ, Park SJ, Seok YJ, Kim P, Lee JH, Lee KH, Cha SS. 2011. FrsA functions as a cofactor-independent decarboxylase to control metabolic flux. Nat Chem Biol 7:434-436*.*

4. Simon R, Priefer U, Pühler A. 1983. A broad host range mobilization system for *in vivo* 76 genetic engineering: transposon mutagenesis in gram negative bacteria. Nat Biotechnol 1:784-791.

5. Keen NT, Tamaki S, Kobayashi D, Trollinger D. 1988. Improved broad-host-range plasmids for 78 DNA cloning in Gram-negative bacteria. Gene 70:191-197.

6. Jeong HS, Jeong KC, Choi HK, Park KJ, Lee KH, Rhee JH, Choi SH. 2001. Differential expression of *Vibrio vulnificus* elastase gene in a growth phase-dependent manner by two different types of promoters. J Biol Chem 27:13875-13880.

7. Kim HS, Park SJ, Lee KH. 2009. Role of NtrC-regulated exopolysaccharides in the biofilm formation and pathogenic interaction of *Vibrio vulnificus*. Mol Microbiol 74: 436-453.

8. Milton DL, O’Toole R, Hörstedt P, Wolf-Watz H. 1996. Flagellin A is essential for the virulence of *Vibrio anguillarum*. J Bacteriol 178:1310-1319.
