# Supplementary material for: Transition of Dephospho-DctD to the Transcriptionally Active State via Interaction with Dephospho-IIAGlc
Source: mBio. 2022 Mar 21;13(2):e03839-21. doi: 10.1128/mbio.03839-21 (PMC9040800; doi:10.1128/mbio.03839-21)
Supplement: TABLE S2 [file mbio.03839-21-st002.docx]

**Table S2. Oligonucleotides used in this study**

**Strain/Gene Primer name Sequences** ^a^ **(5'->3'; Restriction sites underlined)**

**For construction of deletion mutants**

Δ*glpK*  GlpK-upF CCGCTCGAGGCACCGCGTGGAGCCATGAACCCACTGTTG

GlpK-upR AACTGCAGTTTACCCAGTGCTTCAACGAGTGTAGAGCT

GlpK-downF AACTGCAGGCACTGGGCGCTGCGTACCTAGCAGGCTTGGCC

GlpK-downR GCTCTAGAGCCACCGCTTCTGGTGTAGTGCCGATATCA

**For construction of site-directed mutagenized mutants** ^b^

*dctD*_D57E_ DctDmt-F GATCGGGCCCATGGACAAGTGGTATTTCTCGAT

DctDmt-R GATCGAGCTCTCAGCTGTCGGTGTAATCGGCGCG

D57E_AgeI-F GCGATCAAAACCG*GTG*AGTTTGACGGTATC

D57E_AgeI-R GATACCGTCAAA*CTC*ACCGGTTTTGATCGC

*dctD*_D57Q_  D57Q_AgeI-F GCGATCAAAACCG*GTC*AGTTTGACGGTATC

D57Q_AgeI-R GATACCGTCAAA*CTG*ACCGGTTTTGATCGC

*crr*_H75Q_  Crrmt-F GATCGGGCCCCATGGGTCTGTTTGACAAACTTAAG

Crrmt-R GATCTCTAGATTACTTAGTTAGCGTAGAACTGG

H75Q_SacI-F GACGATGGTGTTGA*G*CT*C*TTTGTT*CAG*TTCGGTATCGACACAG

H75Q_SacI-R CTGTGTCGATACCGAA*CTG*AACAAA*G*AG*C*TCAACACCATCGTC

**For construction of complementation plasmids**

*glpFK*  GlpFK-comF CCCAAGCTTCTGCGGTAGAGCTCAACAGCGAGCTGCAC

GlpFK-comR GCTCTAGAGGCGATTTTAATCTTCGAGATCGCGCAATTCAG

**For construction of plasmids overexpressing recombinant proteins** ^b^

rDctD_D57Q_ DctD-F GGGGATCCATGGACAAGTGGTATTTCTCGATGGATGCAGTTTCTTT

DctD-R GGGGTACCTCAGCTGTCGGTGTAATCGGCGCGATTCAGACC

D57Q-F GCGATCAAAACCGGC*CAG*TTTGACGGTATC

D57R-R GATACCGTCAAA*CTG*GCCGGTTTTGATCGC

rGlpK GlpK-F CGGGATCCATGACTGAGCAGAAGTACATCGTTGCACTGGAC

GlpK-R CCCAAGCTTCGCCCAAAATTGGGGGGAGGCGATTTTAATC

**For construction of Bacterial Two-Hybrid system plasmids**

*dctD* DctD-F(TH) ATGCGGATCCGGACAAGTGGTATTTCTCGATGGATGCAGTT

DctD-R(TH) ATGCGAGCTCTCGTTCGGCAGAAATCAGCTGTCGGTG

*crr* Crr-F(TH) ATGCGGATCCGGAGCATGACACAATGGGTCTGTTTG

Crr-R(TH) ATGCGGTACCCTAAGTAGTAATTACTTAGTTACGCGTAGAAC

^a^ Restriction sites are underlined and their usages in cloning experiments are described in "Materials and Methods".

^b^ Altered nucleotides for mutagenizing are indicated with italicized letters.
